# Influence of Rapid Malaria Diagnostic Tests on Treatment and Health Outcome in Fever Patients, Zanzibar—A Crossover Validation Study

**DOI:** 10.1371/journal.pmed.1000070

**Published:** 2009-04-28

**Authors:** Mwinyi I. Msellem, Andreas Mårtensson, Guida Rotllant, Achuyt Bhattarai, Johan Strömberg, Elizeus Kahigwa, Montse Garcia, Max Petzold, Peter Olumese, Abdullah Ali, Anders Björkman

**Affiliations:** 1 Malaria Control Programme, Ministry of Health and Social Welfare, Zanzibar, Tanzania; 2 Infectious Diseases Unit, Department of Medicine, Karolinska University Hospital, Karolinska Institutet, Stockholm, Sweden; 3 Division of International Health, Department of Public Health Sciences, Karolinska Institutet, Stockholm; 4 Médecins Sans Frontières, Dar es Salaam, Tanzania; 5 World Health Organization (WHO) Country Office, Dar es Salaam, Tanzania; 6 Nordic School of Public Health, Gothenburg, Sweden; 7 Global Malaria Programme, WHO, Geneva, Switzerland; London School of Hygiene & Tropical Medicine, United Kingdom

## Abstract

**Background:**

The use of rapid diagnostic tests (RDTs) for *Plasmodium falciparum* malaria is being suggested to improve diagnostic efficiency in peripheral health care settings in Africa. Such improved diagnostics are critical to minimize overuse and thereby delay development of resistance to artemisinin-based combination therapies (ACTs). Our objective was to study the influence of RDT-aided malaria diagnosis on drug prescriptions, health outcomes, and costs in primary health care settings.

**Methods and Findings:**

We conducted a cross-over validation clinical trial in four primary health care units in Zanzibar. Patients of all ages with reported fever in the previous 48 hours were eligible and allocated alternate weeks to RDT-aided malaria diagnosis or symptom-based clinical diagnosis (CD) alone. Follow-up was 14 days. ACT was to be prescribed to patients diagnosed with malaria in both groups. Statistical analyses with multilevel modelling were performed. A total of 1,887 patients were enrolled February through August 2005. RDT was associated with lower prescription rates of antimalarial treatment than CD alone, 361/1005 (36%) compared with 752/882 (85%) (odds ratio [OR] 0.04, 95% confidence interval [CI] 0.03–0.05, *p*<0.001). Prescriptions of antibiotics were higher after RDT than CD alone, i.e., 372/1005 (37%) and 235/882 (27%) (OR 1.8, 95%CI 1.5–2.2, *p*<0.001), respectively. Reattendance due to perceived unsuccessful clinical cure was lower after RDT 25/1005 (2.5%), than CD alone 43/882 (4.9%) (OR 0.5, 95% CI 0.3–0.9, *p* = 0.005). Total average cost per patient was similar: USD 2.47 and 2.37 after RDT and CD alone, respectively.

**Conclusions:**

RDTs resulted in improved adequate treatment and health outcomes without increased cost per patient. RDTs may represent a tool for improved management of patients with fever in peripheral health care settings.

**Trial Registration:**

Clinicaltrials.gov NCT00549003

*Please see later in the article for Editors' Summary*

## Abstract 2

Anders Bjorkman and colleagues report results from a cross-over trial evaluating rapid diagnostic testing for malaria diagnosis in Zanzibar.

## Editors' Summary

**Background:**

Every year, nearly one million people (mainly children living in sub-Saharan Africa) die because of malaria, a subtropical and tropical parasitic disease. Although several parasites cause malaria, *Plasmodium falciparum* is responsible for most of these deaths. Indeed, infection with *P. falciparum* can be fatal within hours if left untreated. For the past 50 years, the main treatments for *P. falciparum* malaria have been chloroquine and sulfadoxine/pyrimethamine. Unfortunately, parasitic resistance to both of these “monotherapies” is now widespread and the illness and death caused by *P. falciparum* in sub-Saharan Africa and elsewhere has been increasing. To combat this increase, the World Health Organization now recommends artemisinin combination therapy (ACT) for *P. falciparum* malaria in all regions with drug-resistant malaria. In ACT, artemisinin derivatives (new, fast-acting antimalarial drugs) are used in combination with another antimalarial to reduce the chances of *P. falciparum* becoming resistant to either drug.

**Why Was This Study Done?:**

The chances of *P. falciparum* becoming resistant to ACT should also be reduced by giving ACT only to people who definitely have malaria. Unfortunately, many people who do not have malaria are given ACT because symptom-based (clinical) diagnosis cannot always distinguish between patients whose fever is caused by malaria and those who have a different infection and who would, therefore, gain more benefit from other treatments. Microscopic detection of parasites in blood smears would greatly improve the accuracy of malaria diagnosis, but this test is rarely available in rural clinics in developing countries. Might the recently developed “rapid diagnostic tests” (RDTs) for *P. falciparum* provide an alternative way to improve malaria diagnosis and thus reduce the overuse of ACT? In this “cross-over trial,” the researchers investigate the effect of the routine use of an RDT for the diagnosis of malaria on ACT prescribing, health outcomes, and costs in four primary health-care clinics in Zanzibar (part of the United Republic of Tanzania), one of the first regions in sub-Saharan Africa to introduce ACT.

**What Did the Researchers Do and Find?:**

Each clinic used RDT-aided symptom-based clinical diagnosis of malaria (the RDT arm of the trial) and symptom-based clinical diagnosis (the CD arm) in alternate weeks to decide whether patients attending with fever had malaria. ACT was prescribed to everyone diagnosed with malaria; during RDT weeks only patients with positive RDT results were prescribed ACT. During the trial, 36% of the 1,005 patients in the RDT arm were prescribed ACT compared to 85% of the 882 patients in the CD arm. 37% and 27% of the RDT and CD arm patients, respectively, were prescribed antibiotics and fewer RDT-arm patients than CD-arm patients returned to the clinic because they still felt ill. The overall cost per patient was similar in both arms. The researchers also report that 23% of the antimalarial treatments given to patients in the RDT arm and 80% of those given to patients in the CD arm were given to people with no microscopically detectable parasites in their blood. Importantly, none of the 26 patients in the RDT group who had positive smears but who were not treated with antimalarial drugs because of a negative RDT result developed severe malaria.

**What Do These Findings Mean?:**

These findings suggest that the replacement of clinical diagnosis alone with RDT-aided diagnosis may reduce the number of people prescribed ACT who do not have malaria and may increase the number of patients given antibiotics for nonmalarial illnesses without increasing costs. However, while the health-care workers involved in this study only prescribed ACT to those patients in the RDT arm who had a positive RDT result (as stipulated in the trial protocol), evidence from other studies suggests that health-care workers often give antimalarials to patients with negative RDT results. Consequently, these findings may not be generalizable to other clinics. Nevertheless, it is reassuring that none of the patients who had malaria that was detected by blood smear but that was missed by RDT subsequently developed severe malaria. This finding, if replicated, might persuade health-care workers to trust RDT results rather than prescribing ACT to everyone with a fever “just in case.”

**Additional Information:**

Please access these Web sites via the online version of this summary at http://dx.doi.org/10.1371/journal.pmed.1000070.

- This study is further discussed in a *PLoS Medicine* Perspective by Zeno Bisoffi and colleagues
- The MedlinePlus encyclopedia contains a page on malaria (in English and Spanish)
- Information is available from the World Health Organization on malaria (in several languages) and on rapid diagnostic tests for malaria. Their 2008 World Malaria Report includes information about global efforts to control malaria and the latest information on malaria in the United Republic of Tanzania
- The US Centers for Disease Control and Prevention provide information on malaria (in English and Spanish)
- Information is available from the Roll Back Malaria Partnership on its approach to the global control of malaria and on artemisinin-based combination therapies

## Introduction

Morbidity and mortality due to *Plasmodium falciparum* malaria have been increasing in sub-Saharan Africa since the early 1990s, concomitantly with spread of resistance to commonly used monotherapies, i.e., chloroquine and sulfadoxine-pyrimethamine [1], [2]. This increased resistance has necessitated that many African countries change their treatment policy to artemisinin-based combination therapy (ACT) as a first-line treatment for uncomplicated malaria. The restricted use of ACT to confirmed malaria patients is critical. Overuse of the more expensive ACTs will not only put an extra heavy financial burden on malaria control programmes in Africa, but also enhance drug resistance and prevent other causes of fever from being appropriately treated, for example, pneumonias, which require antibiotics.

Symptom-based or clinical malaria diagnosis has proven to be quite unspecific [3]–[7]. Malaria diagnosis based on parasitological confirmation is therefore increasingly advocated. Integrated Management of Childhood Illness (IMCI) algorithms based on clinical symptoms could potentially be made more efficient and cost-effective if simple parasitological diagnostic methodologies were incorporated. The use of microscopy has been tried in various health care settings, but is associated with problems of logistics, sustainability, and quality control [8], [9]. The development of rapid diagnostic tests (RDTs) for *P. falciparum* malaria offers a potential alternative in remote and poorly resourced health facilities that are beyond the reach of high-quality microscopy services [10]–[14]. The combination of RDT and ACT provides an important strategic opportunity to reduce malaria-associated mortality in Africa, and RDT use will potentially improve treatment of other causes of fever, for example, life-threatening bacterial diseases [15], [16]. However, the evidence base is still inadequate for malaria control programmes to recommend the use of RDTs on a large scale. There are several studies on sensitivities and specificities of various malaria diagnostic methods [11]–[14], [16]–[19]. In two recently published studies on the implication of RDT use at the health facility level on drug prescription, both describe major problems with test efficiency when used in clinical practice [15], [20]. However, this may be attributed to different messages regarding the risk of withholding malaria treatment to patients with negative test results [21]. Also, these studies did not describe staff training on technique and validation of RDTs, a prerequisite for the malaria diagnostic tests to become cost effective [22]. Furthermore, and importantly, there are no randomized control trials on the health impact and cost-effectiveness of confirmatory malaria diagnosis based on RDTs [18].

Zanzibar was among the first regions in sub-Saharan Africa to introduce ACT, free of charge through public health care, as both as first- and second- line treatment for uncomplicated malaria, which are provided free of charge through public health care. In view of the fact that many patients with fever are prescribed ACT without being malaria infected, the present study was undertaken to assess, on a wide scale, the added value of RDT to clinical diagnosis (CD) alone for management of patients of all ages presenting with fever at primary health care facilities. The hypothesis was that RDT-aided diagnosis of fever patients would improve rational use of ACTs and possibly other necessary treatments, such as antibiotics to non-malaria patients, with an overall improved health impact.

## Material and Methods

### Study Area and Study Health Centres

The trial was conducted in four Primary Health Care Units (PHCUs) in Zanzibar, namely, Muyuni and Uzini on Unguja Island, and Kinyasini and Mzambarauni on Pemba Island. The selection of the four study sites aimed to provide a representative picture of Zanzibar with regard to malaria epidemiology as well as previous use of RDT in Zanzibar. By the time of the trial, malaria transmission in Zanzibar was generally considered endemic [23], with recorded malaria parasite rates between 10% and 50% in different age groups (unpublished data, Zanzibar Ministry of Health). A previous clinical trial conducted in two comparable PHCUs had shown an overall malaria parasite prevalence of about 30% among febrile children aged <5 y [24]. Two PHCUs (Muyuni and Mzambarauni) had been trained in and used RDT for malaria diagnosis 1 y before the study, as part of a Médecins Sans Frontières–supported programme in several districts of Zanzibar; the other two PHCUs (Uzini and Kinyasini) had, up to the time of the study, provided malaria treatment based on CD only. Beside that there were no differences in staffing or general health care capacity between the four PHCUs and they were all considered representative of rural PHCUs in Zanzibar. Because of possible heterogeneity among PHCUs statistical multilevel methods were used in the analyses. Further, to reduce the potential bias on the patient level a cross-over design was introduced within each PHCU.

### Pre-study Training of Nurses

As in other PHCUs in Zanzibar, registered nurses, with 3 to 4 y of formal training, were responsible for providing out-patients care in all study sites. All nurses involved in the study had previously been trained in malaria case management, i.e., diagnosis and treatment including the IMCI algorithm, by Malaria Control Programme and District Health Management Teams of Ministry of Health. In addition and prior to the study start the staff members of all four health facilities received a one-day training on the use of RDTs according to the manufacturer's instructions. This training covered performance of the test and interpretation of the result. Specific instructions were also given to the nurses to consider other treatments and referral especially in children with RDT-negative results and to encourage their guardians or mothers to come back with their children if fever persisted or condition deteriorated. The study design was described in detail to the staff. The nurses were responsible for study implementation. The nurses received a salary supplementation of Tanzanian Shillings (TSh) 25,000 (equivalent of USD 23) per study week. Their ordinary salary was equivalent to about USD 136 at the time of the trial.

### Study Design

This was a nonrandomized four-centre clinical trial but with weekly cross-over validation comparing CD plus RDT (CD+RDT) versus CD alone. Each health facility alternately used CD+RDT or CD alone on a weekly basis. Two sites were allocated to CD+RDT the first week of the trial and the other two to CD alone. During the CD-alone weeks RDTs were not to be used. During RDT weeks the study nurses were encouraged to give antimalarial treatment based on the RDT results, i.e., to prescribe ACT only to RDT-positive patients. They were visited by a member of the research team at least once weekly.

The study was conducted in accordance with Good Clinical Practice [25] and and the Zanzibar Health Research Council declared the work as part of malaria control interventions and polices in Zanzibar. Informed consent was obtained from participants or parents/legal guardians of enrolled children. The study is registered at http://www.clinical.trials.gov with study identification NCT00549003.

### Patient Enrolment and Treatment

The study was conducted during two periods in 2005: 15 February to 15 April, and 30 May to 10 August. These periods thus included both low and peak seasons for malaria transmission in Zanzibar. All patients of all ages with a past history of fever within last 48 h and symptoms compatible with uncomplicated malaria were eligible and screened for the study. Other inclusion criteria included provision of informed consent and living within the catchments area of the health facility. Patients were not enrolled if previously included in the study or if the study nurse was not present at the health facility at the time of attendance.

Treatment of uncomplicated malaria was prescribed according to national policy in Zanzibar: artesunate with amodiaquine as first-line treatment and artemether with lumefantrine as second-line treatment. Drug intake was unsupervised. Patients with severe manifestations of malaria or any other danger signs were required to be referred to the next level of care for further treatment. Study nurses were required to prescribe other treatments in accordance with the IMCI guidelines and their general clinical judgement.

### Patient Follow-up

All enrolled patients were issued a prescription book in which clinical findings and prescribed medications were to be recorded during a 14-d follow-up.

Patients were instructed to return for assessment at any time if symptoms persisted or deteriorated, but also if symptoms recurred during the 14-d follow-up period. All patients were also asked to report back to the health facility routinely 14 d after enrolment. Patients were to be actively followed up if they did not report back within 14+2 d. Upon the study completion, each participant then received one insecticide-treated net free of charge.

If a patient made an unscheduled visit between days 1 through 13 due to fever, the study nurse was to prescribe new treatment(s) based on the diagnostic tool available (CD+RDT or CD alone) at time of reattendance or to refer the patient. Patients developing symptoms of severe malaria were to be referred to the next level of health care. On the scheduled day 14 follow-up visit, patients were asked to report their perceived health status and intake of any concomitant medicines during follow-up. Concomitant medications were to be recorded in the patient case record form, regardless of whether the patient returned to the health facility of enrolment or attended any other health facility.

### Malaria Diagnosis

CD of uncomplicated malaria was generally based on presence or history of fever and absence of clear symptoms indicating alternative causes of fever, e.g., otitis, acute respiratory tract infection, etc. For children below age 5 y, CD was based on the IMCI algorithm. We used a histidine-rich protein (HRP) 2 based test, Paracheck Pf (Orchid Biomedical Systems, India), as RDT. The RDT result was provided to the patient in support of the treatment prescribed.

Thick blood smears (BSs) were collected from all patients at enrolment, on the scheduled day 14 follow-up visit, and at any unscheduled reattendance at the health facility during follow-up days 1 through 13. All BSs were brought to a central laboratory and independently examined by two qualified microscopists, reader 1 (R1) and reader 2 (R2), after staining with 3% Giemsa according to standard procedures [26]. Parasite density per microlitre (µl) of blood of positive BSs was estimated by counting asexual malaria parasites against 200 to 500 white blood cells (WBCs), assuming a presence of 8,000 WBCs per µl of blood [26]. A total number of 100 high-power microscope fields were examined before concluding a negative BS. Discordant BS results between R1 and R2, i.e., positive versus negative or with more than 50% difference in parasite densities, were subjected to examination by a third reader (R3). In the analyses, parasite presence and geometrical mean (GM) densities were based on R1 and R2 readings for concordant slides, and on the two most similar readings for discordant slides. Results of the enrolment BS were routinely provided to the patients on the day 14 follow-up visit, or at reattendance due to other illness.

### Statistical Analyses

According to initial sample size calculations, a total of 850 participants were needed to identify an assumed reduction in malaria diagnosis of 50% with the addition of RDT to clinical diagnosis, at a 5% significance level and a power of 80%, without controlling for design effect of clustering within PHCUs. However, during the initiation of the trial the Ministry of Health and Social Welfare requested that the study be powered also for secondary outcomes. A higher number of participants were thus included in the trial, i.e., up to 1,887. The design effect was not initially accounted for in the power calculation but was accounted for in the final statistical analyses by multilevel modelling. Multilevel analysis with regard to weeks was not applied since observed slight seasonal variation is not based on weekly differences.

Patient data were recorded in case report forms. The final BS, see above, was regarded as the gold standard in the analyses of sensitivity and specificity for the respective diagnostic procedures. Data were entered in Microsoft Excel and analyzed in Epi Info version 3.3.2 and Stata 10. Two-level models with sites (PHCUs) as second level clustering were applied. Odds ratios (ORs) with 95% CIs and associated Chi-square tests and *p*-values (two-sided) were calculated, where applicable. Statistical significance was defined as *p*<0.05.

## Results

**Figure 1:**
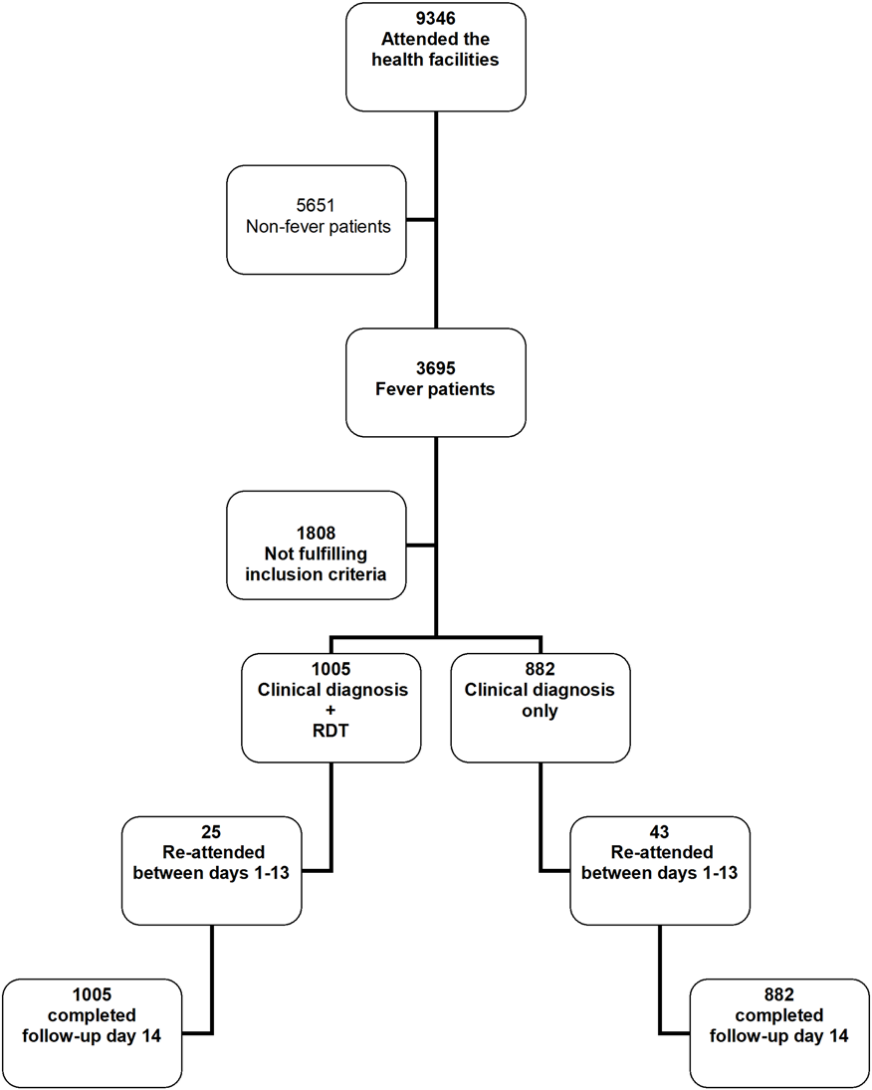
Flow of patients through the trial.

A total of 9,346 patients attended the four health facilities during the study. There was no significant difference in rate of fever among patients attending during the first and second substudy period, i.e. 1,587/4,177 (38%) and 2,108/5,169 (41%), respectively. Of these 3,695 fever patients a total of 1,887 met the inclusion criteria (Figure 1), of whom 1,047 (55%) were children below age 5 y. The main reasons for exclusion were long distance to health facility, previously included in the study, or study nurse not present at the time of attendance.

A total of 1,005 and 882 patients were allocated to the CD+RDT and CD-alone group, respectively. There was a balanced enrolment rate among the four health facilities (range 424 to 528 patients per site). There was a tendency for the number of patients enrolled per week to increase with time, i.e., in parallel with the seasonal increase of malaria transmission during the second study period. However, no systematic difference in patient attendance or enrolment was found between CD+RDT and CD-alone weeks. The study period included an additional CD+RDT week in two health facilities, explaining the higher overall number of patients enrolled in the CD+RDT group. Baseline characteristics (age, sex, number of severe malaria manifestations) were similar in the two study arms (unpublished data). Parasite prevalences were also similar—305/1005 (30%) and 247/882 (28%)—as were mean parasite densities (unpublished data) in the CD+RDT and CD alone groups, respectively.

Diagnostic outcomes, microscopy results, and distribution of antimalarial treatments in the respective arms, and overall microscopy results by age group, are presented in Table 1. Antimalarial medicines were prescribed only to patients diagnosed with malaria in both groups. This restricted prescription of ACT was well perceived by patients and/or their parents. A total of 1,113 patients in the two groups received a prescription of antimalarial drugs on day 0, of whom 1,097/1,113 (99%) were prescribed artesunate+amodiaquine, whereas sulfadoxine-pyrimethamine and quinine was given to six and ten patients, respectively. There were apparent site differences in effect OR (CD+RDT versus CD alone) of prescription rates, and testing for homogeneity confirmed significant differences between sites. Statistical multilevel modelling was thus used. RDT-aided diagnosis was associated with statistically significantly lower prescription of antimalarial treatment, 361/1,005 (36%) compared with 752/882 (85%) in CD alone (OR 0.04, 95% CI 0.03–0.05, *p*<0.001). The rate of antimalarial prescriptions in the CD+RDT group was particularly low (16%) in patients above age15 y, whereas the prescriptions rates were similar in all age categories of CD-alone patients, ranging from 84% to 87% (Table 1).

**Table 1:** Proportions of fever patients diagnosed as malaria by age group according to CD+RDT, CD alone, and microscopy.

Diagnostic Test	Age <5 y	Age 5–15 y	Age >15 y	Total
**CD+RDT**	228/544 (42%)	93/210 (44%)	40/251 (16%)	361/1,005 (36%)
**CD alone**	423/503 (84%)	169/196 (86%)	160/183 (87%)	752/882 (85%)
**Microscopy**	374/1,047 (36%)	128/406 (32%)	50/434 (12%)	552/1,887 (29%)

A majority of antimalarial prescriptions were for children below 5 y, 228/361 (63%) in CD+RDT group and 423/752 (56%) in CD alone group. Prescription in relation to microscopy results are presented in Table 2.

**Table 2:** Proportions of patients receiving antimalarial drugs and antibiotics in relation to day 0 microscopy results.

Drugs Received by Patients	Diagnostic Testing	Blood Slide Result	<5 years	5–15 years	>15 years	Total
**Antimalarials**	CD+RDT	BS positive	186/200 (93%)	71/72 (99%)	22/33 (67%)	279/305 (91%)
		BS negative	42/344 (12%)	22/138 (16%)	18/218 (8%)	82/700 (12%)
	CD alone	BS positive	174/174 (100%)	54/56 (96%)	17/17 (100%)	245/247 (99%)
		BS negative	249/329 (76%)	115/140 (82%)	143/166 (86%)	507/635 (80%)
**Antibiotics**	CD+RDT	BS positive	51/200 (26%)	4/72 (6%)	3/33 (9%)	58/305 (19%)
		BS negative	190/344 (55%)	52/138 (38%)	72/218 (33%)	314/700 (45%)
	CD alone	BS positive	38/174 (22%)	4/56 (7%)	1/17 (6%)	43/247 (17%)
		BS negative	140/329 (43%)	28/140 (20%)	24/166 (14%)	192/635 (30%)

A total of 607/1,887 (32%) patients were prescribed antibiotics, including mainly cotrimoxazole, but also ampicillin, amoxicillin, and erythromycin. Prescription of antibiotics was significantly higher in the CD+RDT than CD-alone group, 372/1,005 (37%) and 235/882 (27%) (OR 1.8, 95%CI 1.5–2.2, *p*<0.001), respectively (Table 2). The majority of antibiotics were prescribed to children below age 5 y, 241/372 (65%) treatments in the CD+RDT group and 178/235 (76%) in the CD-alone group. Concomitant antibiotic and antimalarial treatment was prescribed to 71/1,005 (7%) patients in the CD+RDT compared with 129/882 (15%) in the CD-alone group. Antibiotics alone were prescribed to 301/1,005 (30%) patients in the CD+RDT group compared with 106/882 (12%) in the CD-alone group (OR 3.1, 95% CI 2.4–4.0, *p*<0.001). In the CD+RDT group 343/1,005 (34%) received neither antimalarial nor antibiotic treatment, whereas this occurred in only 24/882 (3%) in the CD-alone group (OR 21.4, 95% CI 13.9–33.1, *p*<0.001).

A total of 1,601/1,887 (85%) patients were prescribed antipyretics, mainly paracetamol but also acetyl salicylic acid. Antipyretics were prescribed to 87.0% versus 82.4% (OR 1.4, 95% CI 1.0–1.9, *p* = 0.037) of patients in the CD+RDT and CD-alone groups, respectively, but in similar proportions to all age groups (CD+RDT 84%–88%, CD alone 79%–84%).

The general microscopy results are presented in Table 1. A total of 552/1,887 (29%) blood slides were positive with a GM density of 3,840 (range 16–457,236 and 95% CI 3,150–4,681) asexual parasites/µl of blood. The results of CD+RDT and CD alone in relation to BS results are presented in Table 3. Sensitivities, specificities, and predictive values of both diagnostic methods are presented in Table 4. Calculated RDT sensitivities were > 99% for detecting a parasite density of ≥1,000 parasites/µl, 76% and 59% for parasite densities 100–999 and <100 parasites/µl, respectively, and thus generally 94% for ≥100 parasites/µl.

**Table 3:** RDT and CD in relation to blood slide results in all age groups combined.

Diagnostic Result	Blood Slide Positive	Blood Slide Negative	Total
**RDT positive**	279	82	361
**RDT negative**	26	618	644
**Total**	305	700	1,005
**CD positive**	245	507	752
**CD negative**	2	128	130
**Total**	247	635	882

**Table 4:** Sensitivities, specificities, and positive and negative predictive values in RDT and CD arms, respectively, in relation to blood slide results.

Test Performance	RDT	CD
**Sensitivity**	92%	>99%
**Specificity**	88%	20%
**Positive predictive value**	77%	33%
**Negative predictive value**	96%	98%

Antimalarial and antibiotic prescriptions in relation to age and BS results are presented in Table 2. Among a total of 552 BS-positive patients, 28 (14 children below age 5 y) were not prescribed antimalarial treatment, 26 after CD+RDT (RDT negative), and two after CD alone. Their parasite densities at enrolment were, however, relatively low (GM 174 parasites/µl blood, range 32–2029). Among patients with BS negative results a total of 82/700 (12%) were prescribed antimalarial drugs in the CD+RDT group (RDT positive) compared with 507/635 (80%) in the CD-alone group (Table 2). A total of 82/361 (23%) antimalarial treatments in the CD+RDT group and 507/635 (80%) in the CD-alone group may thus have been unnecessary according to microscopy results (Tables 1 and 2). In contrast, BS-negative patients received significantly more antibiotics in the CD+RDT group, 314/700 (45%) patients compared with 192/635 (30%) in the CD alone group (OR 2.1, 95%CI 1.6–2.6, *p*<0.001) (Table 2).

The majority (1,813/1,887 [96%]) of patients returned for the scheduled follow-up visit between 14 and 16 d after enrolment. The remaining 74 patients were actively followed up by the research team within three weeks. No deaths occurred during the study. A total of 68 patients returned to the health facility because of ill health during the 2-wk follow-up, mostly (51/68 [75%]) within the first week. Of these 68 patients, 52 were children below age 5 y, nine were 5–15 y old, and seven were adults. The rate of reattendance due to perceived unsuccessful clinical cure was significantly lower in the CD+RDT (25/1005 [2.5%]) than in the CD-alone group (43/882 [4.9%]) (OR 0.5, 95% CI 0.3–0.9, *p* = 0.005).

The BS collected at the time of reattendance were negative in 65/68 (96%) of these patients. The three positive slides, all from the CD-alone group, had low densities: 64, 80, and 1,866 parasites/µl of blood. All three patients had initially been prescribed ACT.

The health outcomes in patients with incorrect malaria diagnoses, both false negative and false positive, are of particular importance. Out of 28 patients (26 in CD+RDT and two in CD-alone groups) with positive BS and not treated with antimalarial drugs, only one returned because of any illness during the two-week follow-up, carrying a low density of 32 parasites/µl of blood. On day 14, an additional four of 28 patients were found to be still BS positive (32, 85, 360, and 47,065 parasites/µl). Among 549 patients having negative BS but clinically diagnosed and treated for malaria, only 20 (3.6%) returned to the health facility with persistent symptoms.

Of 1,113 patients treated for malaria in both groups, 42 (3.8%) returned with illness, compared with 26/773 (3.4%) who did not receive antimalarial treatment. Among patients with positive BS on day 0 and treated for malaria, the frequencies of positive slides on routine follow-up between days 14 and 16 were similar in the two groups, 39/279 (14%) in the CD+RDT and 38/245 (16%) in the CD-alone group. The overall malaria treatment failure after nonsupervised drug intake was thus 77/524 (15%) on day 14, with a GM of 611 (range 16–34,500) parasites/µl.

The mean total costs per patient by age group are presented in Table 5. They include costs of transport, consultation, RDT, and treatment at study inclusion and at reattendance during follow-up period because of illness. Overall, adding RDTs became cost saving in patients above15 y, when malaria treatment is reduced to less than 20% of fever patients.

**Table 5:** Mean total costs (USD) per patient by age group in CD+RDT and CD alone groups, respectively.

Group	Costs	<5 y	5–15 y	>15 y	All ages
**CD+RDT group**	General costs	1.90	1.90	1.90	1.90
	Drugs	0.39	0.63	0.69	0.51
	Reattendance	0.08	0.06	0.03	0.06
	Total mean costs	2.37	2.59	2.62	2.47
**CD alone group**	General costs	1.40	1.40	1.40	1.40
	Drugs	0.58	0.89	1.55	0.85
	Reattendance	0.17	0.05	0.05	0.12
	Total mean costs	2.15	2.34	3.00	2.37

All estimates are based on an exchange rate of USD 1 = TSh 1,100. General costs = transport (USD 0.90)+consultation (USD 0.50)+RDT (USD 0.50) = USD 1.90. Drugs = ACT (USD 0.50−1.40)+antibiotics USD (0.30−0.90)+antipyretics (USD 0.05−0.20). Reattendance costs = transport (USD 0.90)+consultation (USD 0.50)+drugs (ACT, antibiotic+antipyretics = average USD 1.10) = USD 2.50.

## Discussion

We found an overall 2-fold reduction in prescription of antimalarial drugs and reattendance of patients due to illness during the two-week follow-up period in the CD+RDT group compared with CD-alone group. Overall costs were, however, similar in the two groups despite a significant reduction of cost among the adult patients after RDT-aided diagnosis.

Almost all enrolled fever patients in the CD-alone arm were considered and treated as malaria patients, resulting in high diagnostic sensitivity (99%) but low specificity (20%). This result follows the suggestion that fever alone may be a better criterion for malaria treatment than more complicated algorithms [4]. Studies on clinical diagnostic algorithms have shown that with weighting and scoring systems for clinical signs and symptoms may result in sensitivities of 70%–88% and specificities of 63%–82% [3], [4], [17], [27]. However, these methods may be too complicated to be effective under operational conditions, and the algorithms may be site- and context-specific [4].

Health workers learnt to use RDTs correctly with relative ease, confirming that the tests are simple to perform and interpret [11]. The estimated sensitivity (>100 parasites/µl of blood) is in line with WHO recommendations [10] and is also in accordance with a recent review concluding that the accuracies of the HRP2-based test in *P. falciparum–*endemic areas are normally high with a mean sensitivity of 93% [13]. The specificity in our study—88%—was similar to or lower than in some previous studies [12], [13], [15], [16], [19]. Especially under field conditions, heat and time stability could be an important impediment for the optimal use of RDTs for malaria, but according to the manufacturer Parachek Pf is expected to be stable at temperatures up to 40°C for up to two years.

The use of confirmatory malaria diagnosis with RDT is expected to reduce the overuse of antimalarial drugs by ensuring that treatment is targeted to patients suffering from malaria infections as opposed to treating all patients with fever. Our findings confirm this expectation, although the impact of RDT-aided diagnosis will obviously be highly dependent on the malaria incidence (prevalence of malaria in fever patients) in a given situation.

Importantly, in our study, the study nurses showed great confidence in the RDT results as a guide to choice of treatment, as did the patients. This is in contrast to the assumption that care providers, although willing to perform diagnostic tests, do not always comply with the results, especially when the result is negative [15], [20]. High adherence by prescribers in relation to RDT results was, however, also reported in a recent study conducted in mainland Tanzania [28]. We believe that the high compliance and confidence in the RDT in our study may result from a successful pre-study training, although local beliefs, behaviours, and treatment traditions may also account for discrepancies between our results and those of previous publications [15], [20]. We further realise that the study situation, supervision, and incentives provided to the nurses may also affect compliance, but we do not believe it has seriously biased our results. The incentive to the nurses was consistent with common practice for project participation in Zanzibar, but whereas it represented up to approximately a 65% increment of the ordinary salary it was not influenced or affected by performance. Our results obviously need to be confirmed before RDT can be more generally recommended, but we do believe they suggest that RDT use may be efficient if local diagnostic and treatment traditions are properly addressed.

Fearing false negative test results and being aware that delays in providing effective treatment can be fatal for malaria patients is reported to be the main reason to prescribe antimalarial drugs despite a negative RDT result. Importantly, in our study, the patients with malaria detected by BS but non-detectable by RDT and therefore not treated with antimalarial drugs had relatively low parasite densities and no patients developed any severe malaria manifestations during the two week follow-up. This supports a general recommendation of consistence in not treating RDT negative patients. Re-testing will, however, obviously be required if the illness remains or aggravates.

Our finding of a reduction in perceived illness during a two-week follow-up in the CD+RDT group of patients is critical. This was probably attributed to improved treatment of patients with fever not associated with malaria. More antibiotics were prescribed to the RDT-negative patients. The introduction of RDT and ACT thus provides an opportunity to improve the treatment of both malaria and bacterial diseases.

We did consider the potential selection bias of the four health facilities; indeed, significant heterogeneity was observed with regard to the primary effect parameter. We do, however, assume this heterogeneity was at least partly accounted for by multilevel analysis and, since the RDT effect on drug prescriptions was quite large in each PHCU, it seems unlikely to be due to selection bias.

The selection of the four study sites was done to provide a relatively representative picture of both malaria epidemiology and previous use of RDT in Zanzibar. Since RDTs had already been introduced by MSF in some parts of Zanzibar we opted for including PHCUs both with previous experience (two sites) and without previous RDT use (two sites). Beside the previous RDT exposure, the selection of the four study sites was based on representing a common rural situation and representing both of the two major islands in Zanzibar, i.e. Unguja and Pemba (two PHCUs on each island). However, we do of course acknowledge that the choice of the four sites remains arbitrary and of low number and thus cannot be fully representative of an overall Zanzibar situation and even less so of an overall situation in sub-Saharan Africa, which indeed is very diverse itself with regard to epidemiology of malaria, cultural and behavioural aspects, health care structure, etc.

With the understanding that four PHCUs is a very low number, we used a cross-over design of RDT versus non-RDT weeks within sites. The choice of RDT or non-RDT the first week was based on an allocation with one previous MSF/RDT site being in either arm and one non previous MSF/RDT similarly being in either arm. Still, we acknowledge that there may still be confounding effects with regard to health-seeking behaviour or even selection bias by study nurses on respective weeks by (a) patients/caretakers postponing health care attendance to a week with RDT or staff applying exclusion criteria on a CD week and instead request the patient to return on a RDT week, and/or (b) attending alternate PHCUs where RDT is performed. However, we assume that (a) is less realistic, considering that uncomplicated malaria requires urgent treatment and patients or their caretakers as well as health care workers are therefore not likely to wait and postpone treatment. We also do not believe (b) is realistic because systematic RDT use was not implemented outside study PHCUs at the time of the trial and the study sites were located far from each other. And indeed statistical analysis showed no significant difference between frequencies of fever patient attendance on RDT and non-RDT weeks. The only trend observed with regard to frequency of attendance was a tendency to a relative increase toward the later period of the study, compatible with increased malaria transmission.

In summary, RDTs were well performed in peripheral health facilities with acceptable sensitivity and specificity for identifying malaria-attributable fever episodes. The RDT results were adhered to and did provide consistent and significant reduction in antimalarial treatment in parallel with an increase in prescribed antibiotics. This probably contributed to the significant reduction in reattendance due to illness during the two-week follow-up. Our results indicate that RDTs may represent an important tool for improved management of fever patients in peripheral health care settings in malaria-endemic areas, especially where ACT has been introduced for treatment of uncomplicated malaria.

## Supporting Information

Text S1 Trial protocol.
(0.15 MB DOC)

Text S2 CONSORT checklist.
(0.06 MB DOC)

## Abbreviations

ACT: artemisinin-based combination therapy
BS: blood smear
CD: clinical diagnosis
CI: confidence interval
GM: geometrical mean
ICMI: Integrated Management of Childhood Illness
OR: odds ratio
PHCU: Primary Health Care Unit
R: reader
RDT: rapid diagnostic test
WBC: white blood cell
