# Supplementary material for: Influence of Rapid Malaria Diagnostic Tests on Treatment and Health Outcome in Fever Patients, Zanzibar—A Crossover Validation Study
Source: PLoS Med. 2009 Apr 28;6(4):e1000070. doi: 10.1371/journal.pmed.1000070 (PMC2667629; doi:10.1371/journal.pmed.1000070)
Supplement: Text S1 — Trial protocol. (0.15 MB DOC) [file pmed.1000070.s001.doc]

**Title: Efficacy and cost effectiveness of malaria diagnosis procedures and the rational use of ACT in Zanzibar**

**Acronym: ZRDT**

**Updated on:** January, 2005

**Design**: Multi center comparative validation study

**Study** **sites**: Muyuni, Uzini, Kinyasini and Mzambarauni PHCU’s, Unguja & Pemba Islands

# Study director: Dr. Guida Rotllant, MSF

#

**Principal**

**Investigator:** Mr. Mwinyi I. Msellem (HLSO), ZMCP

**Co Investigator** Dr. Montserrant Garcia (MD) - MSF

# Field

# Coordinators: Ms. Narea Lopetegui, MSF, Ms Tahia Rashid ( MLT) MOHSW, Mr. Haji Khatib (HLSO), ZMCP.

# Technical

# Advisors: Prof Zul Premji, MUCHS

# Mr Abdullah S. Ali, ZMCP

# Dr Johan Strömberg, ZAMRUKI

# Dr Oscar Bernal, MSF Barcelona

# Prof Anders Björkman, Karolinska Institutet

# Study Monitor: Dr. E. Kahigwa WHO -TZ

# Data

# Management: ZAMRUKI and ZMCP

# Sponsors: MSF Barcelona, WHO AFRO, Italian Cooperation

# 1 INVESTIGATORS………..……………………………………………………[3](#__RefHeading___Toc470522968)

2 List of abbreviations [4](#__RefHeading___Toc470522969)

3 Introduction [5](#__RefHeading___Toc470522970)

3.1 The malaria situation in Zanzibar [6](#__RefHeading___Toc470522971)

4 Study protocol [6](#__RefHeading___Toc470522975)

4.1 Problem statement [6](#__RefHeading___Toc470522976)

4.2 Study objective [6](#__RefHeading___Toc470522977)

4.3 Design, subjects and methods [7](#__RefHeading___Toc470522978)

4.3.1 Overall study design [7](#__RefHeading___Toc470522979)

4.3.2 Inclusion criteria [7](#__RefHeading___Toc470522980)

4.3.3 Exclusion criteria [7](#__RefHeading___Toc470522981)

4.3.4 Sample size [7](#__RefHeading___Toc470522982)

4.3.5 Locality [7](#__RefHeading___Toc470522983)

4.3.6 Subject recruitment [7](#__RefHeading___Toc470522984)

4.3.7 Duration of study [7](#__RefHeading___Toc470522985)

4.3.8 Bliding procedure ………………………………………………8

4.3.9 Randomization [8](#__RefHeading___Toc470522992)

4.3.10 Concomitant drug therapy [8](#__RefHeading___Toc470522993)

4.4 Medical assessment and blood sampling [8](#__RefHeading___Toc470522994)

4.4.1 Sampling for determining parasitemia 9

4.4.2 Sample labeling ………………………………...…………………….9

4.5 Adverse events [9](#__RefHeading___Toc470523005)

4.5.1 Adverse event definition [9](#__RefHeading___Toc470523006)

4.5.2 Serious adverse event definition [9](#__RefHeading___Toc470523007)

4.5.3 Recording of adverse events [9](#__RefHeading___Toc470523008)

4.5.4 Reporting of serious adverse events [9](#__RefHeading___Toc470523009)

4.6 Bioanalytical assays [10](#__RefHeading___Toc470523010)

4.7 Efforts to control investigational procedures [10](#__RefHeading___Toc470523013)

4.7.1 Monitoring [10](#__RefHeading___Toc470523014)

4.7.2 Audits and inspections [10](#__RefHeading___Toc470523015)

4.7.3 Training [10](#__RefHeading___Toc470523016)

4.8 Data management and evaluation [10](#__RefHeading___Toc470523017)

4.8.1 Data management [10](#__RefHeading___Toc470523018)

4.9 Ethical requirements [11](#__RefHeading___Toc470523020)

4.9.1 Declaration of Helsinki and ethical review [11](#__RefHeading___Toc470523021)

4.9.2 Subject information and consent [11](#__RefHeading___Toc470523022)

4.9.3 Changes to protocol and related procedures [11](#__RefHeading___Toc470523023)

5 References [11](#__RefHeading___Toc470523024)

**6 BUDGET…………………………...………………………………………15**

**Annex I: Laboratory Standard Operating Procedure**

**Annex II: Consent form (Swahili version)**

Annex III: Case Record Form (including Adverse Events Form

**Annex IV: Time frame**

# Investigators

Principal investigator: Mr. Mwinyi I. Msellem (HLSO)

Co-investigator: Dr. Montserrant Garcia (MD)

Study Coordinators: Ms. Narea Lopetegui, Ms Tahia R, Mr. Haji K.

# Technical Advisors: Prof. Z. Premji, Prof. A. Björkman, Mr A. Ali, Dr Johan S, Dr Oscar B.

# Study Monitor: Dr. E. Kahigwa

#### Responsible organizations/Institutes:

**Zanzibar Malaria Control Programme**

Ministry of Health and Social Welfare

P.O. Box 236

Zanzibar – Tanzania

**Medecins Sans Frontieres**

P.O.Box 2185

Zanzibar – Tanzania

**World Health Organisation**

P.O.Box 9292

Dar es Salaam -Tanzania

**Zanzibar Malaria Research Unit Karolinska Institutet**

P.O.Box 2887

Zanzibar – Tanzania

##### Muhimbili University College of Health Sciences

Department of Parasitology

P.O.Box 65001

Dar es Salaam – Tanzania

**Karolinska Institutet**

Malaria Research Unit, Department of Infectious Diseases

Karolinska University Hospital

171 76 Stockholm, Sweden

Universidad Autónoma de Barcelona

Barcelona, Spain

# List of abbreviations

ACT Artemisinin Combination Therapy

AL artemether-lumefantrine (Coartem®)

ASAQ artesunate + amodiaquine

CMM Morbidity Mortality Cohort Study

CRF Case Record Form

HF Health facilities

HLSO Health Laboratory Scientific Officer

IPT Intermittent Presumptive Treatment

ITN Insecticide treated nets

KI Karolinska Institutet

MD Medical Doctor

MLT Medical Laboratory Technician

MOHSW Ministry of Health and Social Welfare

MSF Medecins Sans Frontieres

PPV Predictive Positive Value

PNV Predictive Negative Value

Q quinine

RDT Rapid Diagnostic Test

SP sulphadoxine-pyrimethamine

WHO World Health Organization

ZAMRUKI Zanzibar Malaria Research Unit Karolinska Institutet

ZHRC Zanzibar Health Research Council

ZMCP Zanzibar Malaria Control Programme

ZMoHSW Zanzibar Ministry of Health and Social Welfare

# Introduction

The spread and intensification of antimalarial drug resistance has forced many countries in Sub-Saharan Africa to monitor or change their treatment policies. Zanzibar is implementing Artemisinin based Combination Therapy (ACT) for the treatment of *P. falciparum* malaria since September 2003. The first line drug for uncomplicated malaria in this new policy is artesunate +amodiaquine (ASAQ), and the second line is artemether-lumefantrine ((AL) Coartem®) - quinine (Q) remains the first line drug for treatment of severe malaria.

Although showing increasing failure rates, sulphadoxine-pyrimethamine (SP) is, for reasons of safety, currently the only available alternative for Intermittent Presumptive Treatment (IPT) and therapeutic treatment during pregnancy.

Malaria is the leading public health problem in terms of morbidity and mortality in the Isles. More than 60% of patients presenting at

Government public health facilities are diagnosed as malaria cases. Due to a shortage of resources such as microscopes, electricity, reagents and laboratory practitioners, an estimated 90% of all malaria treated cases in the public health system are based on clinical diagnosis alone. Furthermore, according to a recent ACT effectiveness study (CMM-study, ZAMRUKI), this practice results in an over diagnosis/treatment of malaria exceeding 50%.

There are so many factors which facing developing countries on setup of Malaria microscopy, such as expensively of equipment and reagents and the number of laboratory practitioners is limited in the Isles. However, with the introduction of ACTs the optimization of malaria diagnosis and the rational use of these more expensive new drugs are considered of high importance. Theoretically, accurate malaria diagnosis should reduce the number of patients unnecessarily treated with antimalarials and the number of patients returning to the health facilities because of misdiagnosis, thus reducing the cost of treatment and, equally important, minimizing the potentially resistance driving selection pressure on the parasites.

MSF is currently implementing a malaria project in the South and North A district of Unguja and Wete district of Pemba, Zanzibar. The aim of the project is to improve clinical malaria case management by using ACT in combination with RDT aided diagnosis in health facilities which have no malaria microscopy.

In June 2004 MSF started an intervention in Unguja South and North A district and Wete district in Pemba within Zanzibar archipelago. The project aims at improving malaria case management; supporting the implementation of the new therapy (arteminsin + amodiaquine) and improving the management of anaemia, lower acute respiratory infections and diarrhoea among under 5 year-old children. The time framework is 18 months.

Nurses issue around 80% of the prescriptions delivered, clinical officers issuing the remaining 20%. When the prescribing person in charge is not present in the HF, non-medical staff members (hospital orderlies) carry out the consultation.

Checks of registration books in the health facilities within South District revealed that, since the new protocol started being implemented in September 2003, there had been a transitional period (from September 2003 to January 2004) when the treatment guideline was not followed consistently.

Rapid diagnostic Test (RDT), based on the detection of antigen derived from malaria parasites, represent a good alternative to microscopy for the diagnosis of *Plasmodium falciparum* malaria (WHO, 1966), in areas lacking resources such as electricity, trained laboratory practitioners and supplies of reagents.

The Para check Pf (dipstick and device) was considered as the most appropriate for the use in the field, being sensitive (97%), moderately specific (88%), easy to use and cheap (about US$ 0.5/test), *Guthmann et.al Trans. of the Royal Soc. TMH (2002) 96,254 -257*

The aim of this study is to evaluate the effect of RDT on the accurate malaria diagnosis and rational use of ACT.

## The malaria situation in Zanzibar

Malaria is a major public health problem in Zanzibar. The parasite prevalence in children between the ages of 2 to 11 years is considerable (up to 60 %). Malaria transmission is stable throughout the year. Resistance to chloroquine and sulfadoxine-pyrimethamine is approximately 60%, and 13% respectively.

# Study protocol

## Problem statement

Approximately 90% of all malaria cases are diagnosed in Public Health facilities, using a clinical approach based on signs and symptoms, resulting in an estimated over-diagnosis rate of 40-50%. With the relatively higher cost of ACT and the inherent risk of promoting resistance development by the resulting over-prescription, the cost effectiveness and reduction of overall antimalarial drug pressure expected by introducing RDT diagnosis needs to be scientifically documented.

## Study objective

The primary objective is to compare the proportions of malaria treatments prescribed between two cohorts of clinical diagnosis + RDT and clinical diagnosis alone*.*

Secondary objectives include evaluation of sensitivity, specificity, PPV and PNV of RDT, as well as cost benefit calculations based on reduction of both initial treatment and of revisits due to misdiagnosis.

The selection of the study sites will enable an evaluation of the impact of MSF case management intervention since two out of four sites will already have received “on the job training” on clinical diagnosis through MSF.

## Design, subjects and methods

### Overall study design

## A multicentre randomized validation study comparing RDT + clinical diagnosis versus clinical diagnosis alone in the management of uncomplicated malaria.

### Inclusion criteria

## Any patient attending the study site and reporting history of fever within past 48 hours or other symptoms compatible with malaria and giving their informed consent, will be eligible for the study.

### Exclusion criteria

Patient’s refusal to consent will disqualify participation in the study.

### Sample size

## A total of 850 patients will be enrolled. The number of patients needed in each group, based on the assumed reduction in malaria diagnosis of 50% with the addition of RDT to clinical diagnosis, is calculated to 103, at 95% confidence interval and a power of 80%. With 4 study sites this equals 824 patients. Since no active follow up is intended no margin for drop -outs is necessary.

## 4.3.5 Product used

## Paracheck Pf, Cat No. 30301025, M.L.No 635/L, Lot No. 21071, Mfg. Date May 2004, Exp. Date April 2006, Manufactured by Orchid Biomedical Systems, M 46/47, Phase III B, Verna Industrial Estate, Verna, Goa 403 722, INDIA.

### Locality

## The study will be conducted in four different Health facilities (HF) in Zanzibar Islands, two in each Unguja and Pemba Islands. The tentative HFs are: Muyuni (Unguja South District), Uzini (Unguja Central), Kinyasini (Pemba, Micheweni District) and Mzambarauni (Pemba, Wete District). Each study site will enrol patients into both study arms.

### Subject recruitment

Subjects will be recruited from patients presenting at the study site fulfilling the criteria for inclusion.

### Duration of study

Each patient will spend approximately 1 hour at the study site. There will be a passive follow up period of a minimum of 14 days after the initial visit, during which any subsequent treatment will be evaluated if the patient is seen/examined at the site again. Depending on the rate of enrollment, which is expected to be 100 patients/week/site, the study will run for approximately 45 days in each site.

### Blinding procedure

No blinding.

### Randomization

Each study site will alternately use either RDT+CD or CD alone on a weekly basis (*see Annex V)*. During the CD alone week(s) no RDTs will be available at the site. It will be the same clinician who performs both clinical and RDT aided diagnoses, so as to minimize user dependency. The cycle of RDT+CD/CD alone will be timed in such a way that neighboring sites will not be synchronized, so as to insure stable attendance rates.

### Concomitant drug therapy

Substance, dose and time of administration for any medication (including traditional remedies) taken during FUP shall, if possible, be recorded in the CRF.

## Medical assessment and blood sampling

- - 1. **Clinical assessment**

The Clinician in charge of each Health facility will instruct all patients to come back for follow up assessment any time if symptoms persist or the condition deteriorates, and always on day 14 (or at any convenient time after day 14). At the time of final follow up patients will receive one insecticide treated net (ITN) free of charge.

4.4.2 Sampling for determining parasitemia

A few drops of blood will be taken from all enrolled patients to determine parasitemia: for confirmation of parasitemia by standard microscopy examination in central laboratory in clinical and for the RDT in the intervention arm. The results of RDT will be provided to the patients before treatment, but the microscopy results will only be available for study analysis purposes. Manufacturer’s Instructions Procedure and a special training session by study supervisor prior to study initiation will assist the study clinical officer to perform Rapid Diagnostic Tests. Blood films will be stained with 3% Giemsa for 30 minutes and asexual parasite density will be calculated by counting 200 white blood cells, assuming the white blood cell count of 8000/mm^3^. If less than 10 parasites are detected per 200 white blood cells, estimates will be made against another 500 white blood cells.

### Sample labeling

Each sample (RDT and blood slides) will be labeled by using a sharpened pencil the following information:

- Day of sampling eg day 0 (D0)
- the Patient Code consisting of an abbreviation of the study site eg UZ, MY etc. followed by serial number starting with 1
- subject initials

D0

UZ 003

KIM

091106

- date of sampling.

## Adverse events

### Adverse event definition

An adverse event is an unintended, unfavorable sign, symptom, change in laboratory test, disease, or complaint, temporally associated with the use of the pharmaceutical products in the study, whether or not a causal relationship can be established. Note that the definition could include reasons for administration of concomitant medication or clinically relevant changes in laboratory variables. It could also include reasons for referral to a consultant or unscheduled admission to hospital.

### Serious adverse event definition

A serious adverse event is an adverse event that results in death or is life threatening, an event that requires hospitalization, results in persistent or significant disability or incapacity.

### Recording of adverse events

Any adverse event (AE) encountered during the course of the study will be reported in the Adverse Events Form.

The intensity of an adverse event will be determined according to the following definitions:

*mild* acceptable, easily tolerated by the patient

*moderate* troublesome (inconvenience in performing normal activities?)

*severe* unacceptable (impossible to perform daily routines?)

*unknown*

The adverse events will also be categorized according to if there is a likely causal relationship between the event and the medical products.

*probably* with respect to time sequence of administration of the drug, unlikely to be attributed to concurrent disease or other drugs or chemicals, and which follows a clinical reasonable response on withdrawal

*possibly* but could be explained by concurrent disease or other drugs or chemicals

*unlikely* with a temporal relationship to the drug administration which makes a causal relationship improbable, and in which other drugs, chemicals or underlying disease provide plausible explanations.

### Reporting of serious adverse events

If not present at the study site the principal investigator will immediately be informed by phone or fax of any serious adverse events that occur during the study. In addition to the patient’s CRF a separate detailed case report will be prepared. All serious events will be reported, whether or not considered causally related to the study drug. The principal investigator will ensure that all information documented is submitted to the Ministry of Health, Zanzibar.

## Bioanalytical assays

Blood slides will be collected from all patients at the field and examination will be done centrally at ZMCP offices in Unguja and Pemba Islands

## Efforts to control investigational procedures

### Monitoring

The principal investigator, the study coordinator and field supervisors will ensure that the facilities for conducting the tests remain acceptable and will ensure that the results of the study are being recorded accurately in the CRFs. There will be no formal monitoring.

### Audits and inspections

Not applicable.

### Training

The principal investigator will ensure that appropriate training relevant to the study is given to Clinician to be able to collect the specimens for further investigation and be well familiarized with the procedure and interpretation of the results of RDT. Any information of relevance to the performance of this study is to be forwarded to the co-investigators and other staff involved.

## Data management and evaluation

### Data management

Data will be entered and managed in a secure computer environment at the ZMCP and ZAMRUKI offices. Data analysis will be performed by ZAMRUKI.

## Publications

Each party shall have the right to publish and disseminate information derived from the performance of work. Qualification for authorship shall be in keeping with generally accepted criteria.

ZAMRUKI/KI shall provide the ZMCP manager with a copy of any proposed publication derived from this study for review prior to submission.

Publications shall carry appropriate acknowledgement of funding support by a statement such as the following: *“This publication was supported financially by organisation X….., its contents are solely the responsibility of the authors and do not necessarily represent the official views of the organisation”.*

## Ethical requirements

### 4.9.1 Declaration of Helsinki and ethical review

The study will be performed in accordance with the principles stated in the declaration of Helsinki. The protocol, including the subject information and informed consent form, will be approved by the Ethics Committee at ZHRC, before enrollment of any subjects into the study. The opinions of the committees should be dated and in writing.

All correspondence with the ethics committees will be filed by the Principal Investigator, who is responsible for informing the committees of any serious adverse events and/or major amendments to the protocol.

### Subject information and consent

The investigator will ensure that each subject, and parent/guardian is given thorough verbal and written information about the nature, purpose, and possible risks of the study. The subjects and their parents/guardians will be informed that they are free to discontinue their participation in the study at any time. The principal investigator is responsible for ensuring that signed and witnessed verbal informed consent is obtained from all subjects and/or parent/guardians before enrollment. Written and verbal information will be given in Kiswahili.

### Changes to protocol and related procedures

No change in the investigational procedures shall be effected without the mutual agreement of the study investigators. All changes must be documented by signed protocol amendments.

# References

Massaga, J.J., Kamugisha, M.L. & Salum, F.M. (1999) Malaria presumptive

treatment in Tanzania: Is it a rational approach for malaria management in

rural health units? *African Journal of Health Sciences* **6**: 22-26**.**

Rooth, I.B. & Björkman, A. (1992) Fever episodes in a holoendemic malaria area of Tanzania: parasitological and clinical findings and diagnostic aspects related to malaria. *Trans. Royal Soc. Trop. Med. Hyg* **86**:479-482.

Schellenberg, J.R.M., Smith, T., Alonso, P.L. & Hayes, R.J. (1994) What is malaria?: Finding case definition for field research in highly endemic areas*. Parasitol. Today* **10**: 439-442.

World Health Organization. Anonymous. A rapid dipstick antigen capture **assay for** the diagnosis of falciparum malaria. WHO, Informal Consultation on Recent Advances in Diagnostic Technique and Vaccines for malaria. Bull World Health Ogran 1996; 74:47-54. References

Kahigwa E, Schellenberg D, Schellenberg JA, Aponte JJ, Alonso PL, Menendez C. Inter-observer variation in the assessment of clinical signs in sick Tanzanian children. Trans R Soc Trop Med Hyg. 2002; 96(2):162-6

**6. Budget:**

**Staff allowances**

2 Supervisors x 70,000/- per week x 8 weeks = 1,120,000/-

2 Laboratory technicians x 50,000/- per week x 8 weeks = 800,000/-

4 Prescibers x 50,000/- per week x 8 weeks = 1,600,000/-

4 Community mobilizers x 25,000/- per week x 8 weeks = 800,000/-

1 Data entry clerks x for 4 weeks = 50,000/-

1 Statistician x 150,000/- = 150,000/-

**Sub Total = 4,520,000/-**

**Laboratory Reagents and other consumable materials**

850 Rapid Diagnostic Tests @600/- per test = 510,000/-

Immersion oil 25ml/bottle x 5,000/- x 10 bottles = 50,000/-

50bxs Microscope slides (76x 25mmfrosted edge) pack of 72x3, 000/- = 200,000/-

Slide carrier (vertical) 30 boxes x 10,000/- = 300,000/-

Methanol (2.5ltrs per bottle) @ 50,000/- x 4 bottles = 200,000/-

Glycerol (2.5 ltrs per bottle) @ 38,000/- x 4 bottles = 152,000/-

20 boxes blood lancets (*pack of 200pcs)* x 5,000/- = 100,000/-

10 rolls Cotton wool (500gm) x 2,500/- per roll = 25,000/-

10 Examination gloves x 4,000/- per box = 40,000/-

10 Liters Methylated spirit @8,000/- = 80,000/-

10tins (25 gms) Giemsa stain powder @ 5,000/- = 50,000/-

8 bottles Xylene (500ml.) @ 5,000/- = 40,000/-

1 binocular Microscope, Olympus CX20 BIMF200 = 1,023,000/-

**Sub Total = 2,260,000/-**

**Stationary (Unguja and Pemba)**

## Photo copying and binding works = 103,000/-

5 rim papers @5,000/- = 25,000/-

1 box black point pens = 5,000/-

8 Counter books@ 3,500/- = 28,000/-

10 box files@3,500/- = 35,000/-

1 rim flip charts@10,000/- = 10,000/-

**Sub Total = 300,000/-**

**Incentive for the patients**

850 ITN x 3,000/- = 2,550,000/-

**Sub Total = 2,550,000/-**

#### Travel costs

Flights tickets ZNZ/PBA/ZNZ = 540,000/-

Flight tickets DSM/ZNZ/DSM = 480,000/-

Night allowances = 450,000/-

**Sub total = 1,470,000/-**

**Transportation**

Renting of field vehicle 30,000/- x 6 trips /week x 8 weeks = **1,950,000/-**

**GRAND TOTAL = 13,050,000/-**

*Annex I:*

## STANDARD OPERATING PROCEDURES (SOP) FOR BLOOD SLIDE COLLECTION AND EXAMINATION

- Laboratory Requirements
- Blood examination for parasites

**Laboratory Requirements:**

- Frosted blood slides
- Cotton wool
- Sterile blood lancets
- Wooden dry rack
- Sharpened pencil
- Examination gloves
- 75% methylated spirit
- Record form or register

**Blood Examination for Parasites:**

- Preparation of thick films

- Labeling of films

- Staining of blood smears

- Examination of films

- Storage of films

**Preparation of thick films:**

- Clean finger with cotton swab soaked in antiseptic (methylated spirit). - Dry with dry cotton wool

- Puncture ball of finger with sterile lancet
- Apply gentle pressure and collect 3 drops (about 1.5 μl) adjacent to each other.
- Using the corner of another slide quickly spread drops of blood to form an evenly spread film of ≅ 1 cm in diameter.
- Make two identical films.
- Dry in air or using a hair dryer.
- Label on the frosted edge using a pencil.
- Protect from flies and dust.
- Good thick films should be thin enough to allow a print to be read through.
- Label on frosted end with lead pencil.
- Stain promptly to avoid autofixation

**Staining thick films:**

Stain blood films using the standard method (30min by 3% Giemsa)

**Examination:**

- Count no. of asexual parasites against WBC until 200WBC.
- Complete counting parasites and WBC in the last field even if 200 WBC have been counted, report number of parasites against number of WBC counted.
- If, after 200WBC have been counted, 9 or fewer parasites have been counted, continue counting until you reach 500 WBC, Complete counting parasites and WBC in the last field even if 500 WBC have been counted, report number of parasites against number of WBC counted.
- The number of parasites relative to the WBC count can be converted to parasites per microliter (ul) of blood by the simple mathematical formular : -

Number of parasite/ul of blood = No. Parasites counted x 8,000

No. of WBC

**Storage of slides:**

- Clean off oil using lens tissue soaked in Xylene

- Pack in special slide boxes or

- Wrap with smooth tissue paper and pack in original pack

- Label boxes clearly (Study site and date)

**Labeling of slides:**

- Day of sampling eg day 0 (D0)
- the Patient Code consisting of an abbreviation of the study site eg UZ, MY etc. followed by 3 numbers starting with 001
- subject initials
- date of sampling.

# *Annex II:*

*STANDARD OPERATING PROCEDURE (SOP) FOR RAPID DIAGNOSTIC TESTS*

Materials and equipment required

- Cotton wool
- Sterile blood lancets
- Sharpened pencil
- 75% methylated spirit
- Record form or register
- Timer
- Individual packaged cassettes
- Examination gloves

**Test procedure:**

*Will depend on the manufacture’s instruction*

*Annex III*

Fomu ya ombi la ridhaa

Mimi, __________________________ (*Jina la mzazi wa mtoto*) nina umri wa miaka 18 au zaidi nina mamlaka kamili wa kumshirikisha/kushiriki_______________________________ _________________________________, (*Jina la mtoto)* kwenye utafiti huu wa **“KUPIMA MALARIA KWA KUTUMIA NJIA YA HARAKA”** Utafiti ambao unaendeshwa na Bwana Mwinyi I.Msellem na wenzake kupitia Kitengo cha Malaria, na nimefahamishwa na nimesoma maelezo ya utafiti huu, nimeona umbile la kazi hii, faida zake, madhumuni yake, muda utakaochukuwa kazi hii, ushiriki katika kazi hii si wa kulazimishwa na nimeona hauna madhara kwa yeyote na yale yote yatakayotokeya ni mambo ambayo hayatarajiwi kwa jinsi nilivyoelewa kwa maelezo niliyopewa na ____________________________ (*Jina la aliyetowa taarifa hiyo kwa mgonjwa)*

Nimepewa nafasi ya kuhoji na kutaka ufafanuzi kwa lolote linalohusiana na utafiti huu, nimehoji, na nimeridhika kwa jawabu nilizopata, ikiwa nitakuwa na suali lolote wakati wote wa kazi hii nitamuona _____________________________ (*Jina la mfanyakazi wa Kituo au Mtafiti Mkuu)* Nimefahamishwa na ninaelewa ya kwamba ninaweza wakati wowote kumuondoa/kujiondoa kwenye utafiti huu iwapo ninataka kufanya hivyo, uamuzi wangu huo hautanipelekea kuadibiwa kwa njia yeyote ile.

**NDIO**

Sahihi ya mzazi/ mlezi/mgonjwa__________________________ Tarehe ______________

Jina la mzazi/mlezi/mgonjwa _________________________________________________

Anuani ua makaazi __________________________________________________

Nambari ya mgonjwa __________________________________________________

Sahihi ya shahidi ______________________________ Tarehe ____________

Jina la Shahidi _____________________________________________________

Annex IV

| **TIME FRAME** | | | | | | | | | | | | | | | | | | | |
| --- | --- | --- | --- | --- | --- | --- | --- | --- | --- | --- | --- | --- | --- | --- | --- | --- | --- | --- | --- |
| **Activity** | **Months** | **FEBRUARY,05** | | MARCH, 05 | | | | APRIL, 05 | | | | MAY, 05 | | | | JUNE, 05 | | | |
|  | **Quarters** | **3rd** | **4th** | **1st** | **2nd** | **3rd** | **4th** | **1st** | **2nd** | **3rd** | **4^th^** | **1st** | **2nd** | **3rd** | **4th** | **1st** | **2nd** | **3rd** | **4th** |
| One day training for the Study coordinators and Supervisors at Central level on 14th Feb 05 | |  |  |  |  |  |  |  |  |  |  |  |  |  |  |  |  |  |  |
| One day training, conducting study and Closely supervision for Study team at Muyuni PHCU 15th & 16th Feb 05 | |  |  |  |  |  |  |  |  |  |  |  |  |  |  |  |  |  |  |
| One day training, conducting study and Closely supervision for Study teams at Mzambarauni and Uzini PHCUs 17th & 18th Feb 05 | |  |  |  |  |  |  |  |  |  |  |  |  |  |  |  |  |  |  |
| One day training, conducting study and Closely supervision for Study team at Kinyasini PHCU 21st & 22nd Feb 05 | |  |  |  |  |  |  |  |  |  |  |  |  |  |  |  |  |  |  |
| Patients enrolling in all HFs | |  |  |  |  |  |  |  |  |  |  |  |  |  |  |  |  |  |  |
| Data entry at Central level | |  |  |  |  |  |  |  |  |  |  |  |  |  |  |  |  |  |  |
| Analysis | |  |  |  |  |  |  |  |  |  |  |  |  |  |  |  |  |  |  |
| Preliminary technical report | |  |  |  |  |  |  |  |  |  |  |  |  |  |  |  |  |  |  |
